# The Diversity of Anti-Microbial Secondary Metabolites Produced by Fungal Endophytes: An Interdisciplinary Perspective

**DOI:** 10.3389/fmicb.2013.00065

**Published:** 2013-03-27

**Authors:** Walaa Kamel Mousa, Manish N. Raizada

**Affiliations:** ^1^Department of Plant Agriculture, University of GuelphGuelph, ON, Canada; ^2^Department of Pharmacognosy, Mansoura UniversityMansoura, Egypt

**Keywords:** pathogen, mycology, natural products, anti-microbial, antifungal

## Abstract

Endophytes are microbes that inhabit host plants without causing disease and are reported to be reservoirs of metabolites that combat microbes and other pathogens. Here we review diverse classes of secondary metabolites, focusing on anti-microbial compounds, synthesized by fungal endophytes including terpenoids, alkaloids, phenylpropanoids, aliphatic compounds, polyketides, and peptides from the interdisciplinary perspectives of biochemistry, genetics, fungal biology, host plant biology, human and plant pathology. Several trends were apparent. First, host plants are often investigated for endophytes when there is prior indigenous knowledge concerning human medicinal uses (e.g., Chinese herbs). However, within their native ecosystems, and where investigated, endophytes were shown to produce compounds that target pathogens of the host plant. In a few examples, both fungal endophytes and their hosts were reported to produce the same compounds. Terpenoids and polyketides are the most purified anti-microbial secondary metabolites from endophytes, while flavonoids and lignans are rare. Examples are provided where fungal genes encoding anti-microbial compounds are clustered on chromosomes. As different genera of fungi can produce the same metabolite, genetic clustering may facilitate sharing of anti-microbial secondary metabolites between fungi. We discuss gaps in the literature and how more interdisciplinary research may lead to new opportunities to develop bio-based commercial products to combat global crop and human pathogens.

## Introduction

Plant pests and pathogens including viruses, bacteria, nematodes, insects, and fungi reduce crop yields by 30–50% globally, contributing to malnutrition and poverty (Pimentel, 2009). There are 700 known plant viruses, such as cassava mosaic virus (CMV) which devastates the livelihoods of cassava farmers in Africa (Thresh and Cooter, 2005). Amongst bacterial pathogens, blights caused by *Xanthomonas* species cause 350 different plant diseases including rice blight disease (*X. oryzae*) (Leyns et al., 1984). Nematodes are considered significant problems in tropical and subtropical regions, but are often undiagnosed because they exert their damage on plant roots (Shurtleff and Averre, 2000). Nematodes such as those belonging to the genera *Paratrichodorus* and *Trichodorus* are also vectors of plant pathogenic viruses (Boutsika et al., 2004). Approximately 9,000 species of insects and mites damage crops, causing an estimated 14% loss in global crop yields, requiring $13 billion in pesticide controls in the U.S. alone (Pimentel, 2009). Fungi are considered to be particularly serious plant pathogens because they can also potentially produce mycotoxins that are then consumed by humans and animals. For example, the maize and rice pathogenic fungus *Fusarium moniliforme* produces fumonisin B1 which is associated with esophageal cancer (Gelderblom et al., 1991). The maize pathogenic fungus *Aspergillus flavus*, which causes kernel rot, produces aflatoxin on pre-harvest corn and in storage (Payne and Widstrom, 1992). *Fusarium graminearum*, the causal agent of head blight in wheat and ear rot in maize, produces toxic trichothecenes including deoxynivalenol (DON) (Sutton, 1982). Combined, aflatoxin and DON cause $1.5 billion in losses each year across different crops in the U.S. alone (Robens and Cardwell, 2003). Other serious fungal pathogens of crops include: *Magnaporthe grisea* and *Pyricularia oryzae*, which cause rice blast diseases in Asia; *Puccinia* sp., the causal agents of rusts in barley, maize, and wheat; *Phytophthora infestans*, an oomycete fungus which infects the Solanaceae family and led to the potato blight in Ireland in the 1840s; and *Rhizoctonia solani*, the causal agent of damping-off in diverse crops and stem canker in potato (Strange and Scott, 2005).

Synthetic pesticides including fungicides are widely used in pathogen management but there are increasing demands to develop environmentally friendly, bio-based products. Bio-based strategies include the use of biocontrol agents such as plant growth promoting bacteria (Compant et al., 2005), and fungi such as *Trichoderma viridi*, which has been used to control *Rhizoctonia* stem canker and black scurf of potato (Beagle-Ristaino and Papavizas, 1985).

A potential opportunity to control crop pathogens is the use of endophytes and their derived secondary metabolites. Endophytes are microorganisms which inhabit plants in the tissues beneath the epidermal cell layers but cause no apparent harm to their hosts (Stone et al., 2000). In fact, endophytes can confer diverse beneficial traits to host plants (Johnston-Monje and Raizada, 2011a,b). All plants that have been investigated for endophytes possess them (Strobel and Daisy, 2003), and since there are more than 300,000 species of land plants, these microbes represent a large reservoir of biological resources including bioactive compounds with potential applications for medicine, industry and agriculture, including pathogen control.

The objective of this paper is to review the diversity of anti-microbial compounds synthesized by fungal endophytes including terpenoids, alkaloids, phenylpropanoids, aliphatic compounds, polyketides, and peptides. Bioactive compounds in general synthesized by fungi were reviewed from different perspectives (Strobel and Daisy, 2003; Aly et al., 2010). We review compounds from the interdisciplinary perspectives of biochemistry, genetics, fungal biology, host plant biology, human and plant pathology. We conclude by discussing common themes.

## Terpenoid Compounds

Sesquiterpenes, diterpenoids, and triterpenoids are the major terpenoids that have been isolated from endophytes.

### Sesquiterpenes

**Figure 1:**
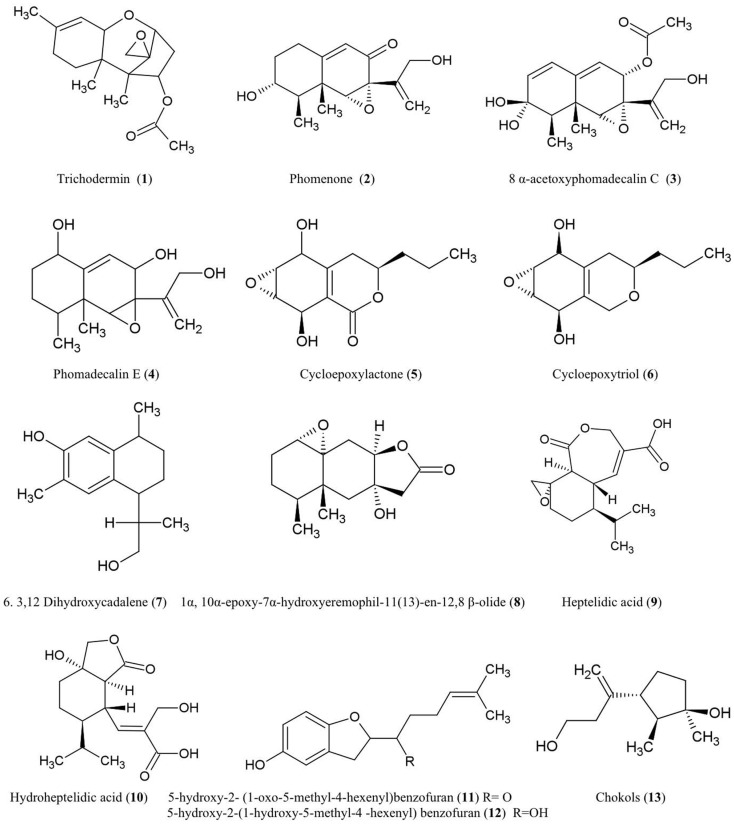
**Structures of sesquiterpene derivatives of fungal endophyte origin (1–13)**.

The structures of sesquiterpene derivatives described in this review are shown (Figure 1).

#### Trichodermin (1)

Trichodermin was characterized from *Trichoderma harzianum*, an endophytic fungus living in *Ilex cornuta*, an evergreen holly shrub from East Asia (Chen et al., 2007). Trichodermin is a member of the 12,13-epoxytrichothecene mycotoxin family which has been used as a template for chemical synthesis of pharmaceutical compounds and plant growth regulators (Cutler and LeFiles, 1978). Trichodermin has been reported to protect against the Solanaceous plant pathogens *Alternaria solani* and *R. solani* *in vitro* (Chen et al., 2007). However, trichodermin has been shown to have inhibitory effects on plant growth including wheat coleoptiles (*Triticum aestivum*), tobacco (*Nicotiana tabacum*), beans (*Phaseolus vulgaris*), and corn (*Zea mays*) (Cutler and LeFiles, 1978). Mechanistically, trichodermin has been shown to be a very potent inhibitor of eukaryotic protein synthesis, specifically by inhibiting peptide-bond formation at the initiation stage of translation (Carter et al., 1976) and by inhibiting peptidyl transferase activity required for translational elongation and/or termination (Wei et al., 1974).

#### Phomenone (2)

The antifungal eremophilane sesquiterpene, phomenone, is produced from *Xylaria* sp., an endophytic fungus associated with *Piper aduncum*, a tree of the pepper family from the New World. Phomenone has been claimed to have antifungal activity against *Cladosporium cladosporioides* (wheat pathogen) and *C. sphaerospermum* (common indoor mold) though supporting evidence was not presented (Silva et al., 2010). Phomenone is also a phytotoxin and is assumed to be a causal agent of wilted leaves in tomato; citing unpublished data, the authors suggested that phomenone induces electrolyte loss and dysfunction of cell membrane permeability (Capasso et al., 1984). Phomenone is structurally similar to the sesquiterpene eremophilane ring system of the PR toxin produced by *Penicillium roqueforti*, which inhibits RNA polymerase and protein synthesis at the initiation step as well as elongation (Moule et al., 1976). Phomenone has been used as a natural precursor for synthesis of anti-cancer ester drugs (Weerapreeyakul et al., 2007).

#### 8α-Acetoxyphomadecalin C (3) and phomadecalin E (4)

These eremophilane sesquiterpenes were obtained from the endophytic fungus, *Microdiplodia* sp. KS 75-1, from the stems of conifer trees (*Pinus* sp.). The compounds showed moderate antibiosis activities on agar assays against the pathogen *Pseudomonas aeruginosa* (ATCC 15442) (Hatakeyama et al., 2010), a standard strain used to evaluate bactericidal disinfectants. Phomadecalin C was also isolated from cultures of *Phoma* sp. (NRRL 25697), a fungus originally isolated from wood decay stromata (Che et al., 2002). Phomadecalin C was shown to be antagonistic to *Bacillus subtilis* (ATCC 6051) in standard disk assays (Che et al., 2002).

#### Cycloepoxylactone (5) and cycloepoxytriol B (6)

These compounds were purified from cultures of the endophytic fungus *Phomopsis* sp. (Valsaceae), isolated from the leaves of *Laurus azorica* (Lauraceae), a laurel tree from the Canary Island of Gomera. Cycloepoxylactone has been shown on agar plates to inhibit the growth of an anther smut fungus (*Microbotryum violaceum*) and a soil inoculant bacterium (*Bacillus megaterium*), while cycloepoxytriol B also inhibited growth of an alga (*Chlorella fusca*) (Hussain et al., 2009).

#### 3,12-Dihydroxycadalene (7)

This was one of five cadinane sesquiterpenes isolated from *Phomopsis cassiae* collected from *Cassia spectabilis* (*Senna spectabilis*), a tree from tropical America belonging to the legume family *Fabaceae*. This compound showed potent antifungal activity against the phytopathogenic fungi *Cladosporium cladosporioides* and *C. sphaerospermum* using a TLC-based assay (Silva et al., 2006).

#### 1α-10α-Epoxy-7α-hydroxyeremophil-11-en-12,8-β-olide (8)

This compound, structurally related to eremophilanolide sesquiterpenes, was obtained from *Xylaria* sp. BCC 21097, isolated from the palm *Licuala spinosa* (Isaka et al., 2010). The compound was active against *Candida albicans* (causative agent of human genital and oral infections) and exhibited activity against the malaria parasite *Plasmodium falciparum* using a microculture radioisotope assay in which failure of the parasite to uptake radiolabeled nucleic acid precursors indicated anti-parasitic activity. The authors hypothesized that this activity may be structurally related to the epoxide moiety (Isaka et al., 2010).

#### Heptelidic acid (9) and hydroheptelidic acid_ENREF_28 (10)

These compounds were isolated from *Phyllosticta* sp., an endophytic fungus inhabiting the needles of *Abies balsamea* (balsam fir tree) from New Brunswick, Canada (Calhoun et al., 1992). In this region, this tree species is affected by defoliating larvae of spruce budworm (*Choristoneura fumiferana*). The endophyte-derived compounds were shown to be toxic to these larvae (Calhoun et al., 1992) which have caused hundreds of millions of dollars in losses to the Canadian forestry sector (Chang et al., 2012).

#### 5-Hydroxy-2- (1-oxo-5-methyl-4-hexenyl)benzofuran (11) and 5-hydroxy-2-(1-hydroxy-5-methyl-4-hexenyl) benzofuran (12)

These two new benzofuran-carrying normonoterpene derivatives were isolated from cultures of an unidentified endophytic fungus obtained from *Gaultheria procumbens*, a ground cover plant that grows between Canadian forest trees infected by spruce budworm, as noted above. These endophyte-derived compounds were found to be toxic to the cells and/or larvae of spruce budworm (Findlay et al., 1997).

#### Chokols (13)

These compounds were isolated from *Epichloë typhina*, an endophyte of *Phleum pratense* (perennial Timothy-grass). The compounds were found to be fungitoxic to the leaf spot disease pathogen *Cladosporium phlei* (Yoshihara et al., 1985).

### Diterpenes

**Figure 2:**
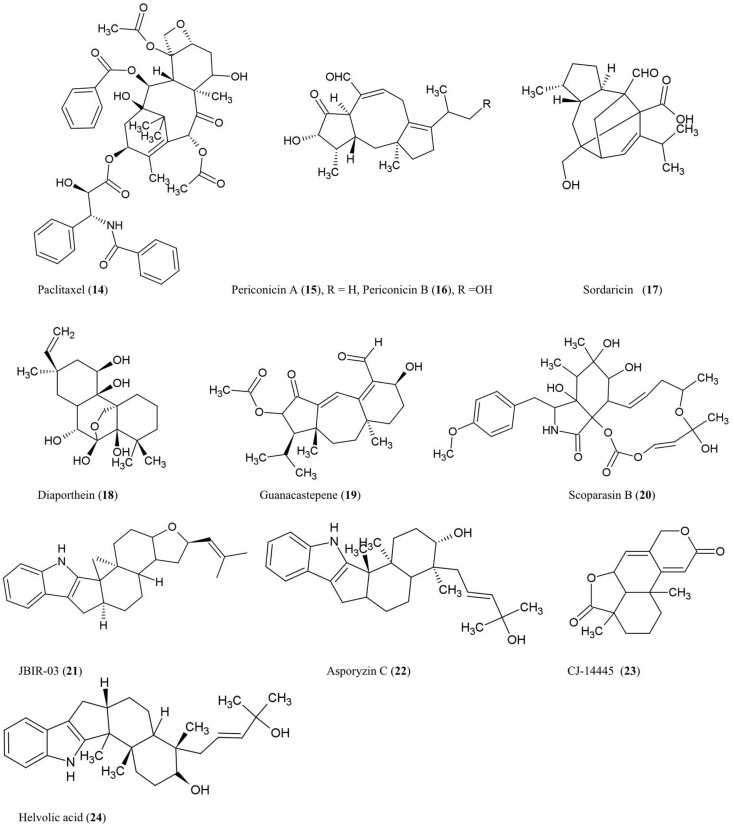
**Structures of diterpene and triterpene derivatives of fungal endophyte origin (14–24)**.

The structures of diterpenes and triterpenes derivatives discussed here are illustrated (Figure 2).

#### Paclitaxel (14)

The anti-cancer and antifungal drug paclitaxel (Taxol) was reported to be produced from the endophytic fungus *Taxomyces andreanae* originally isolated from the inner bark of the yew tree *Taxus brevifolia* in northwestern Montana (Stierle et al., 1993, 1995). Paclitaxel is reported to be produced by ∼20 different endophyte species inhabiting different plant species (Zhou et al., 2010b). Paclitaxel possesses a unique chemical structure composed of a taxane ring with a four-membered oxetane ring and a C-13 ester side chain. Paclitaxel acts by stabilizing microtubules and inhibiting spindle function leading to disruptions in normal cell division (Horwitz, 1994). Paclitaxel is derived from geranylgeranyl diphosphate (GGDP), a compound synthesized from dimethyl diphosphate and indole pyrophosphate, catalyzed by GGDP synthase (Eisenreich et al., 1996). Cyclization of GGDP to taxa-(4,5),(11,12)-diene is catalyzed by taxadiene synthase (TS) which represents the first step in paclitaxel biosynthesis (Hezari et al., 1995). We have independently confirmed the presence of a protein of the expected molecular weight (110 kDa) of TS in a paclitaxel-producing fungal endophyte using a plant anti-TS antibody (Soliman and Raizada, unpublished data). Interestingly, using selective chemical inhibitors and genetic studies, we recently showed that endophytic paclitaxel may be derived from both mevalonate and non-mevalonate pathways (Soliman et al., 2011), a surprising result since non-mevalonate pathway enzymes have only been shown to exist in bacteria and plastids but not fungi. A curiosity remains as to why both fungal endophytes and their host plants produce the same compound, apparently redundantly. Isolation of the genes responsible for fungal paclitaxel biosynthesis may help to reveal whether these two pathways evolved from convergent evolution or parallel evolution.

#### Periconicins A (15) and B (16)

These fusicoccane diterpenes were identified from the endophytic fungus *Periconia* sp. collected from the inner bark of *Taxus cuspidata* by bioassay-guided fractionation (Kim et al., 2004). Periconicin A was found to be a more potent anti-microbial agent than periconicin B against *B. subtilis*, *Klebsiella pneumoniae*, and the opportunistic human pathogen *Proteus vulgaris* (ATCC 3851) using a microtiter broth dilution method (Kim et al., 2004). Periconicin A was also reported to have potent antifungal activity against the human pathogens *C. albicans*, *Trichophyton mentagrophytes* (causative agent of cutaneous infections), and *T. rubrum* (causative agent of jock itch, athlete’s foot and ringworm) (Shin et al., 2005). Periconicins having the same carbon skeleton as fusicoccins, which are a group of plant growth regulators resembling the major plant hormone gibberellins (de Boer and Leeuwen, 2012). Though many antifungal compounds have adverse effects on plant growth, fusicoccins have the potential to remove seeds from dormancy, stimulate seed germination, open leaf stomata, and promote plant growth by cell elongation, though these effects vary by crop species and dosage (Muromtsev et al., 1994; Shin et al., 2005; de Boer and Leeuwen, 2012).

#### Sordaricin (17)

This pimarane diterpene is the aglycon of sordarin and was purified from the fungi *Xylaria* sp. isolated from the leaves of *Garcinia dulcis*, a tropical fruit tree of southeast Asia (Pongcharoen et al., 2008). The compound exhibited moderate antifungal activity against *C. albicans* ATCC90028 using an agar diffusion assay (Pongcharoen et al., 2008). *C. albicans* is an important pathogen of human immunocompromised patients. Sordarin was previously shown to inhibit fungal protein synthesis by selectively binding and inhibiting elongation factor 2 (EF-2) which catalyzes ribosomal translocation during translation (Justice et al., 1998). Replacement of the sugar moiety of sordarin with alkyl side chains increased the antifungal activity against yeast in a manner proportional to the lipophilicity of the alkyl side chain (Tse et al., 1998). Introduction of oxime moieties to sordarin could increase antifungal activity against *C. albicans* and *C. glabrata*, perhaps by altering the spatial orientation of the lipophilic side chain (Serrano-Wu et al., 2002).

#### Diaporthein B (18)

This pimarane diterpene was purified from the culture broth of the fungus *Diaporthe* sp. BCC 6140, isolated from unidentified wood in Thailand, and it showed strong inhibition of growth of *Mycobacterium tuberculosis* using a metabolic indicator colorimetric assay (Alamar Blue) (Dettrakul et al., 2003). The bioassay suggested that the ketone group at position C-7 may be important for the antifungal activity (Dettrakul et al., 2003). This compound may hold promise against the global tuberculosis epidemic, which affects nearly 9 million new patients each year [World Health Organization (WHO), 2012].

#### Guanacastepene (19)

This novel diterpenoid, produced by an unidentified fungus CR115 from a branch of the tree *Daphnopsis americana* growing in Costa Rica, was shown to have antibacterial activity against methicillin-resistant *Staphylococcus aureus* and vancomycin-resistant *Enterococcus faecium* (Singh et al., 2000). As the possible mode of action, guanacastepene was reported to damage bacterial membranes (Singh et al., 2000).

#### Scoparasin B (20)

Structurally related to cytochalasins, this diterpenoid was isolated from the endophytic fungus *Eutypella scoparia* PSU-D44 inhabiting the leaves of *G. dulcis*, a tropical fruit tree of southeast Asia as noted earlier. The compound was shown to have antifungal activity against an important skin pathogen, *Microsporum gypseum*, using a hyphal-extension inhibition assay (Pongcharoen et al., 2006).

#### Compound JBIR-03 (21) and asporyzin C (22)

These compounds, belonging to the tremorgenic mycotoxin indoloditerpenes, were identified from *Aspergillus oryzae*, an endophyte of the marine red algae *Heterosiphonia japonica*, a Pacific seaweed which has become invasive to Europe. JBIR-03 exhibited strong insecticidal activity against brine shrimp (*Artemia salina*), while asporyzin C exhibited potent activities against *Escherichia coli* (Qiao et al., 2010).

#### Diterpene CJ-14445 (23)

This compound was isolated from solid cultures of the endophytic fungus *Botryosphaeria* sp. MHF associated with the leaves of *Maytenus hookeri*, a medicinal plant containing the potent antitumor agent maytansine (Yuan et al., 2009). The compound inhibited growth of *C. albicans*, *Saccharomyces cervisiae*, and *Penicillium avellaneum* using standard agar disk diffusion assays (Yuan et al., 2009).

### Triterpenes

#### Helvolic acid (24)

This nordammarane triterpenoid was isolated from the yeast *Pichia guilliermondii* Ppf9 (asexual form is *Candida guilliermondii*) that colonizes the Himalayan lily family medicinal plant *Paris polyphylla* (Zhao et al., 2010). Helvolic acid was reported to exhibit strong antibacterial activity using broth dilution. It was also reported to inhibit spore germination of *Magnaporthe oryzae*, the causative agent of rice blast disease (Zhao et al., 2010), one of the most damaging diseases of rice. In *Aspergillus fumigatus*, genes encoding helvolic acid are clustered together in the sub-telomere chromosome region (Lodeiro et al., 2009), suggesting that the pathway may have been derived from horizontal gene transfer, possibly from bacteria. In *A. fumigatus*, a major human pathogen, evidence was presented that the helvolic acid gene cluster may be transcriptionally regulated by the major virulence-controlling transcription factor LaeA (Bok et al., 2005).

### Steroids

**Figure 3:**
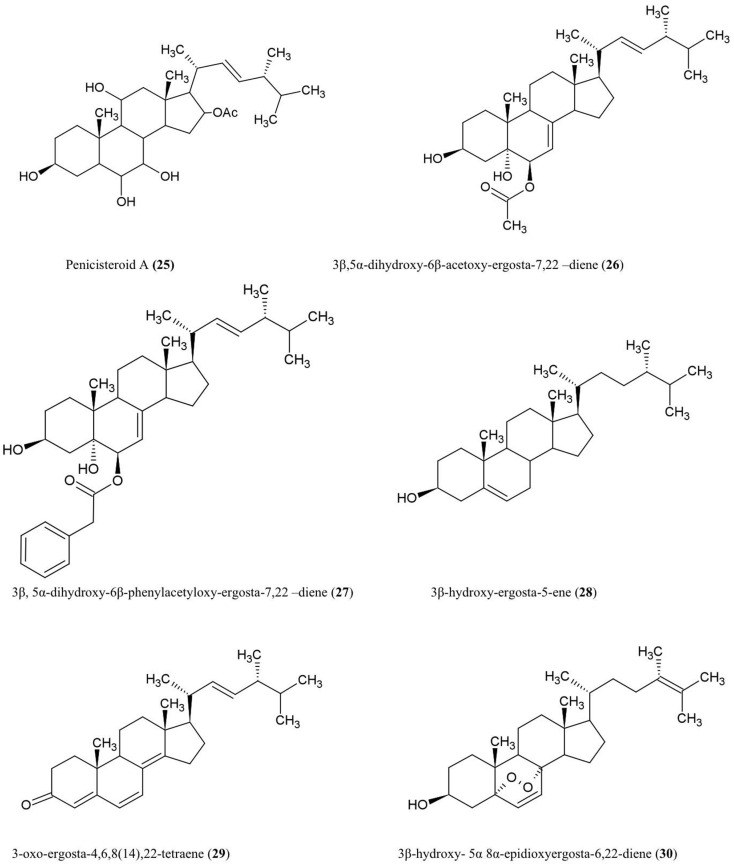
**Structures of additional steroid derivatives of fungal endophyte origin (25–30)**.

Structure of steroids compounds discussed here are illustrated (Figure 3).

#### Penicisteroid A (25)

This steroid was isolated from the culture extracts of a fungus, *Penicillium chrysogenum*, cultured from an unidentified marine red algal species belonging to the genus *Laurencia*. Penicisteroid A showed potent antifungal activity against *Aspergillus niger* (plant black mold) and moderate activity against *Alternaria brassicae* (pathogen of *Brassica* plants such as cabbage) (Gao et al., 2011).

#### Steroids 3β,5α-dihydroxy-6β-acetoxy-ergosta-7,22-diene (26), 3β,5α-dihydroxy-6β-phenylacetyloxy-ergosta-7,22-diene (27), 3β-hydroxy-ergosta-5-ene (28), 3-oxo-ergosta-4,6,8(14),22-tetraene (29), 3β-hydroxy-5α,8α epidioxy-ergosta-6,22-diene (30)

These compounds were isolated from the liquid culture of a fungal endophyte *Colletotrichum* inhabiting the stems of *Artemisia annua* (Lu et al., 2000), a traditional Chinese medicinal herb. The authors reported that all the compounds except (**30**) have antifungal activity against several crop pathogens including *Phytophthora capisici*, *Gaeumannomyces graminis*, *Rhizoctonia cerealis*, and *Helminthosporium sativum*. All of the compounds except (**28**) also showed antibacterial activity against *Pseudomonas* sp, *B. subtilis*, *Sarcina lutea* (*Micrococcus luteus*, a human skin pathogen), and *S. aureus*, and antifungal activity against *A. niger* and *C. albicans* (Lu et al., 2000).

## Alkaloids

**Figure 4:**
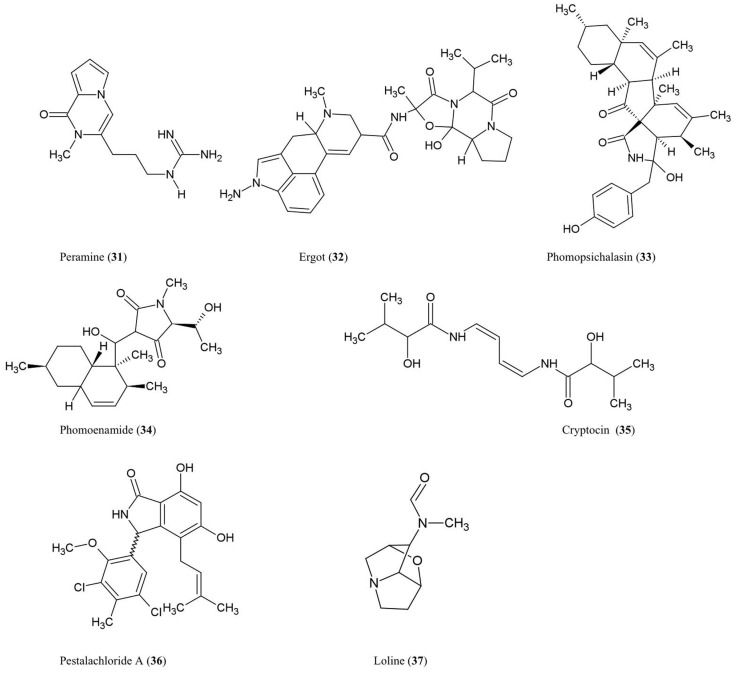
**Structures of alkaloid derivatives of fungal endophyte origin (31–37)**.

The structures of the alkaloidal derivatives described in this review are summarized (Figure 4).

### Amines and amides

#### Peramine (31)

This pyrrolopyrazine alkaloid was characterized from perennial ryegrass (*Lolium perenne* L.) produced by the endophytic fungus *Acremonium lolii* (Rowan, 1993) famous for its production of loline alkaloids (described later). Ryegrass infected with *A. lolii* or purified peramine were shown to be potent anti-feedants of Argentine stem weevil without negatively impacting mammals (Rowan, 1993). Peramine levels were highest in young leaves, and peramine has been identified in a number of grass genera (Rowan, 1993). In Arizona fescue infected with another fungal endophyte, *Neotyphodium*, peramine concentrations were observed to differ between plant genotypes inhabited by the same endophyte haplotype, suggesting that plant genotype plays a major role in regulating this secondary metabolite (Faeth et al., 2002).

#### Ergot alkaloids (32)

These compounds are produced within different species of the grass subfamily Pooideae by sexual *Epichloe* fungi and their asexual derivatives belonging to the genus *Neotyphodium* within the Clavicipitaceae family (Schardl, 2010). These metabolites can act as anti-feedants and/or toxins against insects, nematodes, and mammalian herbivores (Powell and Petroski, 1992; Wallwey and Li, 2011). Related ergot alkaloid-producing fungal parasites (especially *Claviceps purpurea*) of animal grass feed (e.g., tall fescue) have been shown to cause toxicity to livestock, in particular ergovaline (Powell and Petroski, 1992). The first committed step in ergovaline biosynthesis is the prenylation of l-tryptophan with dimethylallylpyrophosphate (DMAPP) to produce 4-dimethylallyltryptophan (4-DMAT), catalyzed by the enzyme DMAT synthase (Heinstein et al., 1971). In total, ergot alkaloid biosynthesis has been shown to involve 14 co-expressed genes which are arranged in a chromosomal cluster in *C. purpurea* (Wallwey and Li, 2011). Seven of these genes are conserved across different fungi and are thought to be responsible for the biosynthesis of the tetracylic ergoline scaffold. Interestingly, in *Epichloe festucae*, the genes for ergovaline biosynthesis were only highly expressed during biotrophic growth of the fungus within the plant not when the mycelia were cultured separately, suggesting that the plant was needed to induce expression of the fungal gene cluster (Wallwey and Li, 2011). For more details about ergot alkaloids, please refer to excellent recent reviews on the subject (Tudzynski et al., 2001; Schardl, 2010; Wallwey and Li, 2011).

#### Phomopsichalasin (33)

This compound is a unique cytochalasan derivative with a novel ring system involving an isoindolone moiety fused to a 13-membered tricyclic system. Phomopsichalasin was isolated from an endophytic *Phomopsis* sp. originating from the twigs of the willow shrub, *Salix gracilostyla* (Horn et al., 1995). Cytochalasans are well known fungal metabolites that can bind actin (Binder and Tamm, 1973) and have been shown to have antibacterial, antifungal, anti-viral, and anti-inflammatory activities (Pendse and Mujumdar, 1986). Phomopsichalasin was shown to be antibacterial against *B. subtilis*, *S. aureus*, and *Salmonella gallinarum* (poultry pathogen), and antifungal against the human pathogenic yeast *Candida tropicalis* (Horn et al., 1995).

#### Phomoenamide (34)

This compound was detected in cultures of an endophytic *Phomopsis* sp. fungus obtained from the leaves of *G. dulcis*, an Indonesian tropical fruit tree. The compound has been shown to have anti-microbial activity against *M. tuberculosis* using the Alamar Blue Assay (Rukachaisirikul et al., 2008).

#### Cryptocin (35)

This tetramic acid analog was purified from the endophytic fungus *Cryptosporiopsis quercina* which inhabits the inner bark of the stems of *Tripterygium wilfordii*, a Chinese medicinal plant used to treat rheumatoid arthritis. Cryptocin has antifungal activity against a wide range of plant pathogens including *P. oryzae*, the fungus causing rice blast disease (Li et al., 2000), one of the most devastating crop diseases worldwide.

#### Pestalachloride A (36)

This compound was purified from *Pestalotiopsis adusta*, an endophytic fungus that inhabits the stem of an unknown Chinese tree; the compound showed antifungal activity against the plant pathogens, *Fusarium culmorum*, *Gibberella zeae* (anamorph *F. graminearum*), and *Verticillium albo-atrum* (Li et al., 2008a). The authors classified this compound as a new chlorinated benzophenone alkaloid belonging to the amine and amide subclass. The authors further noted that pestalachloride A differs significantly from other known alkaloids by having a somewhat rare 2,4-dichloro-5-methoxy-3-methylphenol moiety attached to the isoindolin-1-one core.

### Indole derivative

#### Loline alkaloid (37)

This indole derivative, a saturated 1-aminopyrrolizidine with an oxygen bridge, was detected in the grass *Festuca pratensis* (*Lolium pratense*) originating from its fungal endophyte, *Neotyphodium uncinatum* (Blankenship et al., 2001). Loline has broad-spectrum anti-insect and anti-aphid activity resulting in increased resistance of the host plant against insect herbivores (Wilkinson et al., 2000). A fascinating four-species ecological interaction involving loline was reported, in which the loline-producing endophytic fungus (*N. uncinatum*) inhabiting its host grass protects a non-host plant (*Rhinanthus serotinus*, a parasite of the host grass of the endophyte) against aphids (*Aulacorthum solani*) (Lehtonen et al., 2005). In *N. uncinatum*, two homologous gene clusters encoding loline were identified, named LOL-1 and LOL-2, with LOL-1 containing nine genes within a 25-kb chromosomal segment; as the genes between the two clusters were generally in the same order and orientation, these clusters likely represent relatively recent duplications (Spiering et al., 2005). Based on the identification of these genes, a biosynthetic route for loline was proposed involving the precursors proline and homoserine (Spiering et al., 2005).

*Neotyphodium* is the asexual form, but the sexual derivative, *Epichloe*, can also produce loline. *Epichloe* fungi can be pathogenic to host plant inflorescences and are horizontally transmitted by re-infection, whereas *Neotyphodium* is mutualistic and is transmitted through spores vertically on healthy inflorescences (Schardl, 2010). Expression of the loline biosynthetic genes has recently been used to better understand the pathogenic versus mutualistic forms. In contrast to plants infected by pathogenic *Epichloe* fungus, the fungal loline biosynthetic genes were upregulated in inflorescences of healthy plants inhabited by the mutualistic form (*Neotyphodium*), suggestive of evolutionary selection on the endophyte for increased expression of genes encoding this beneficial insecticide (Zhang et al., 2010).

## Phenolic Compounds

**Figure 5:**
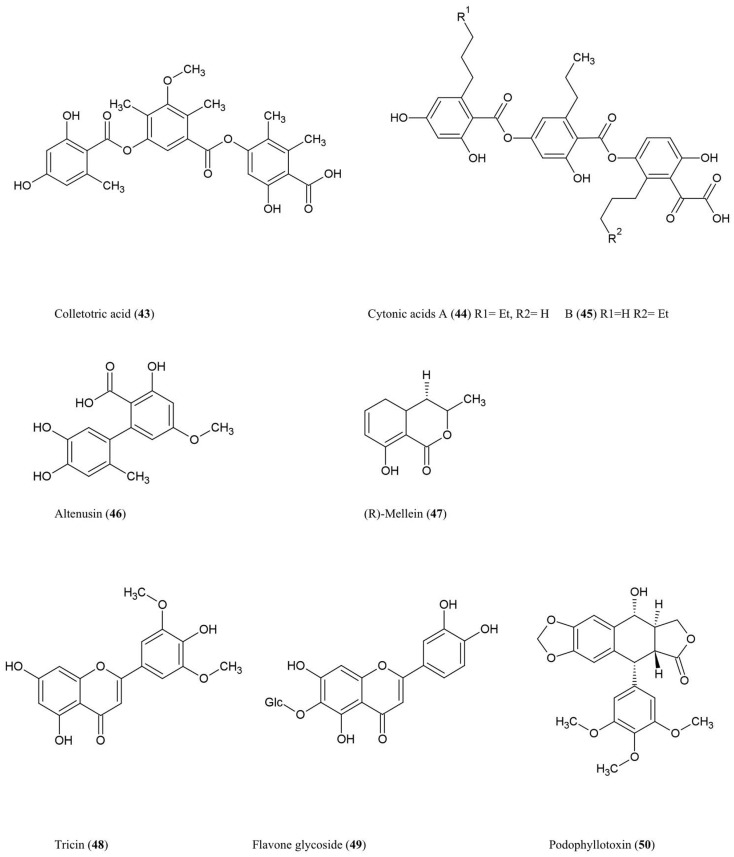
**Structures of phenolic compounds of fungal endophyte origin (43–50) (structures of simple structures 38–42 are not shown)**.

The structures of these phenolic compounds are described below and summarized (Figure 5) (Simple structures **38–42** are not shown).

### Phenols and phenolic acids

#### 2-Methoxy-4-hydroxy-6-methoxymethylbenzaldehyde (38)

This phenolic compound was shown to be antifungal against the cucumber phytopathogen *Cladosporium cucumerinum* using an antifungal-TLC assay. The compound is produced by *Pezicula* strain 553, a fungal endophyte colonizing an unknown tree (Schulz et al., 1995).

#### *p*-Hydroxybenzoic acid (39), *p*-hydroxyphenylacetic acid (40), tyrosol (41), *p*-coumaric acids (42)

These antifungal phenolic acids were purified from the stromata of *Epichloe typhina*, which can be a symptomless endophyte, but can also act as a pathogen against its host *P. pratense* (European Timothy-grass) (Koshino et al., 1988).

#### Colletotric acid (43)

This tridepside compound was characterized from the liquid culture of *Colletotrichum gloeosporioides*, a fungus that colonizes the stems of *Artemisia mongolica*, an Asian plant which shows resistance to insects and pathogens. The compound was shown to have anti-microbial activity against the bacteria *B. subtilis*, *S. aureus*, and *S. lutea* (*M. luteus*), and the fungus *H. sativum* (Zou et al., 2000), the latter being a seedling blight and root rot pathogen of cereals.

#### Cytonic acids A (44) and B (45)

Three novel isomeric tridepsides (*p*-tridepside isomers) were obtained from *Cytonaema* sp., an endophytic fungus of *Quercus* sp. (oak tree). These compounds showed inhibitory activity against the opportunistic human pathogen cytomegalovirus by inhibiting a protease required for normal assembly of the viral nucleocapsid (Guo et al., 2000).

#### Altenusin (46)

This biphenyl fungal metabolite was isolated from *Alternaria* sp. (UFMGCB55), an endophyte of the Asteraceae family plant *Trixis vauthieri*, collected from a natural preserve in Minas Gerais, Brazil (Cota et al., 2008). This plant was investigated because it was known to contain compounds active against the human protozoan parasites *Trypanosoma* and *Leishmania*, which infect millions of people worldwide. Altenusin was shown to inhibit trypanothione reductase (TR), an enzyme required to protect these parasites against oxidative stress, though the compound itself did not diminish parasite viability perhaps due to an inability to traverse intracellular compartments (Cota et al., 2008). Altenusin was reported to have antifungal activity against clinical isolates of *Paracoccidioides brasiliensis*, which causes human Paracoccidioidomycosis, perhaps by inhibiting cell wall synthesis or assembly (Johann et al., 2012).

### Isocoumarin derivatives

#### (R)-Mellein (47)

This isocoumarin was purified from *Pezicula livida* (strain 1156), an endophytic fungus isolated from the European beech tree *Fagus sylvatica* growing in Lower Saxony, Germany. In plate assays, the compound was inhibitory against the bacteria *B. megaterium* and *E. coli*, the fungi *Ustilago violacea* and *Eurotium repens*, and the alga *C. fusca* (Schulz et al., 1995). Mellein and 4-hydroxymellein were also shown to be synthesized by *Aspergillus ochraceus* and showed structural similarity to the dihydroisocoumarin moiety of ochratoxin A, one of the most abundant mycotoxins found in food (Moore et al., 1972). Mullein and 3-hydroxylated derivatives were also isolated from *Botryosphaeria obtusa*, a grapevine pathogen; in grape these compounds are considered potential molecular markers for the presence of pathogenic fungi (Djoukeng et al., 2009).

### Flavonoids

#### Tricin (48) and related flavone glycosides (49)

These flavonoids were isolated from Big Bluegrass (*Poa ampla*), a perennial grass native to Western North America, infected with *Neotyphodium typhnium*, a symbiotic fungus (Ju et al., 1998). However, the compound was not reported from pure fungal cultures. These flavonoids were found to be toxic to the larvae of *Culex pipiens* (Ju et al., 1998), the common house mosquito which can also act as a vector for West Nile Virus in North America. The researchers initially investigated Big Bluegrass for endophytes producing anti-insecticide after observing that endophyte-free plants were more susceptible to spider mites.

### Lignans

#### Podophyllotoxin (50)

This aryl tetralin lignan, first described in 1880, is today an important anti-cancer drug originally isolated from *Podophyllum* plant species in both the Himalayas and North America where indigenous peoples used the plant for medicinal purposes (Stähelin and von Wartburg, 1991). *Podophyllum* has also been shown to have anti-viral and insecticidal properties (Sudo et al., 1998; Gao et al., 2004). More recently, podophyllotoxin was also purified from endophytes inhabiting *Podophyllum* sp. including the fungus *Phialocephala fortinii* (Eyberger et al., 2006). Podophyllotoxin is thus another example of a secondary metabolite produced by both an endophyte and its host. Podophyllotoxin was also obtained from *Fusarium oxysporum*, an endophyte of the medicinal plant *Juniperus recurva* which also originates from the Himalayan mountains (Kour et al., 2008). Podophyllotoxin production was also reported from *A. fumigatus* which is an endophyte of *Juniperus communis* (Kusari et al., 2009). With respect to its anti-cancer activity, podophyllotoxin and its derivatives have been shown to prevent mitosis in late S/early G2 phase by binding to and inhibiting the enzyme (topoisomerase II) required to unwind the double helix of DNA (Canel et al., 2000). The anti-viral activity of Podophyllotoxin appears to be due to its ability to disrupt viral replication and inhibit reverse transcriptase (Canel et al., 2000).

## Aliphatic Compounds

**Figure 6:**
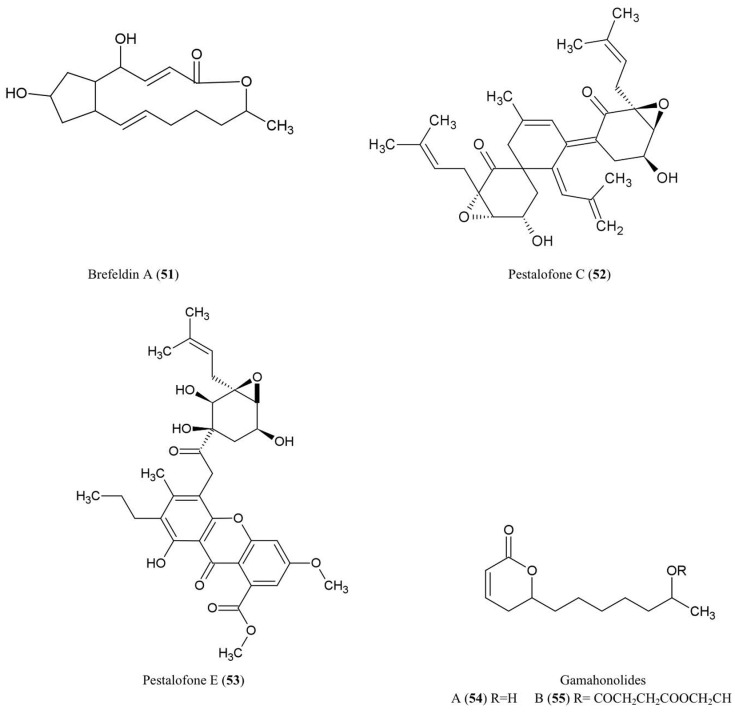
**Structures of aliphatic derivatives of fungal endophyte origin (51–55)**.

The structures of the aliphatic derivatives described in this review are illustrated (Figure 6).

### Brefeldin A (51)

Brefeldin A has become an important research chemical used by cell biologists: Brefeldin A blocks the transport of proteins from the endoplasmic reticulum to the Golgi apparatus resulting in inhibition of secretion (Misumi et al., 1986). An early report showed that this compound could be isolated from cultures of the endophyte *Eupenicellium brefeldianum* (Harri et al., 1963). During the last 50 years, there have been additional reports of endophytes of different hosts producing breveldin A, including *Paecilomyces* sp. and *Aspergillus clavatus*, inhabitants of the conifer trees *Taxus mairei* and *Torreya grandis*, respectively, from southeast China (Wang et al., 2007). Brefeldin A has been shown to have antibacterial, anti-viral, anti-nematode, and antifungal activities (Betina, 1992) including against the fungi *A. niger*, *C. albicans*, and *Trichophyton rubrum* (Wang et al., 2007).

### Pestalofones C (52) and E (53)

These cyclo hexanone derivatives were isolated from cultures of *Pestalotiopsis* *fici*, an endophytic fungus that colonized the branches of an unidentified tree in Hangzhou, China. The compound showed antifungal activity against *Aspergillus fumigates* (Liu et al., 2009). *A. fumigates* causes invasive lung diseases that may cause mortality especially in immune-compromised people.

### Gamahonolide A (54) and B (55)

These compounds were characterized from stromata of *E. typhina* growing on *Phleum pretense* (Timothy-grass). Gamahonolide A showed antifungal activity against *Cladosporium herbarum*, the fungal plant pathogen and common inhalant allergen of humans, using antifungal-TLC guided isolation (Hiroyuki et al., 1992).

## Polyketides

**Figure 7:**
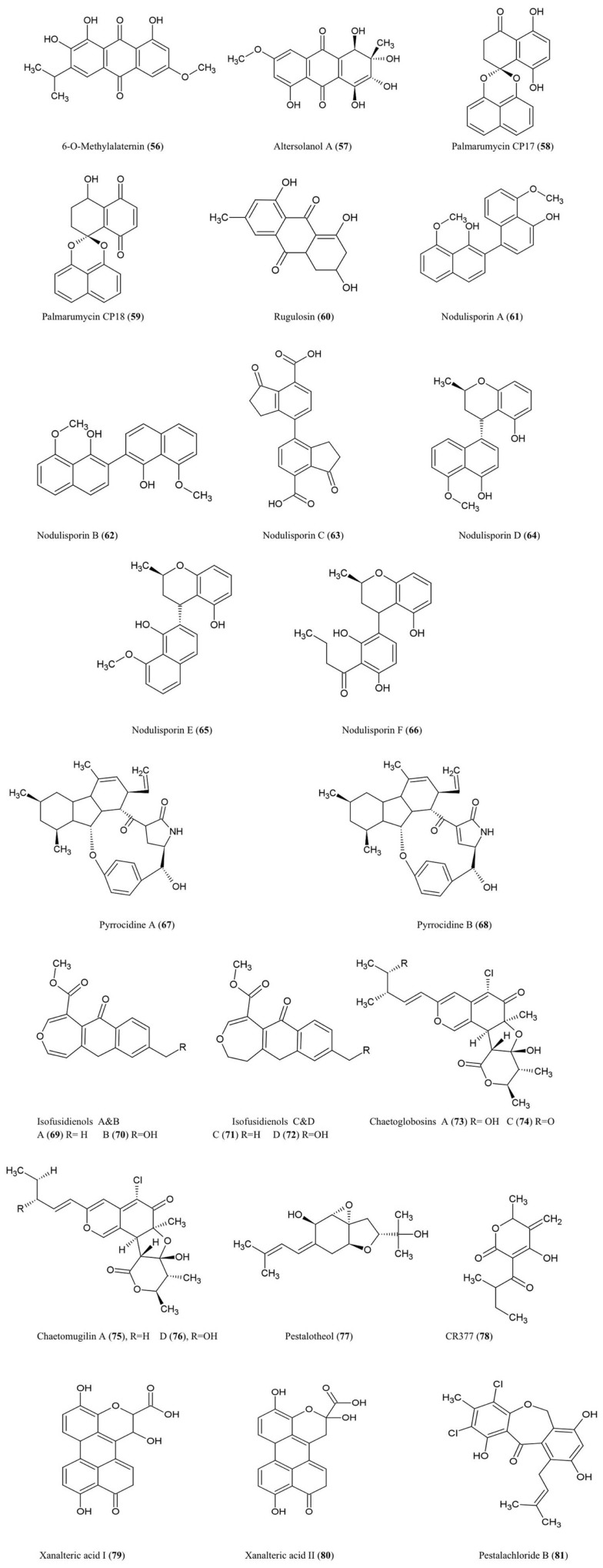
**Structures of polyketide derivatives of fungal endophyte origin (56–81)**.

The structures of the polyketide derivatives noted below are summarized (Figure 7).

### 6-*O*-Methylalaternin (56) and altersolanol A (57)

These tetrahydroanthraquinones were isolated from fungal cultures of *Ampelomyces* sp. (Leptosphaeriaceae), isolated from the medicinal plant *Urospermum picroides* (Asteraceae), collected in Egypt (Aly et al., 2008). *Ampelomyces* were among the first fungi used as biocontrol agents of plant parasitic fungi (Yarwood, 1932). Both quinone compounds exhibited antibacterial activity against *Enterococcus faecalis*, *S. aureus*, and *S. epidermidis* (Aly et al., 2008).

Altersolanol A along with other derivatives were also isolated from the fungus *A. solani* responsible for black spot disease of *Solanum lycopersicum* (tomato) (Okamura et al., 1993). In the latter study, the compound was found to inhibit growth of the bacteria *S. aureus*, *B. subtilis*, *M. luteus*, *E. coli*, and *Pseudomonus aeruginosa* and the fungi, *C. albicans* and *Candida utilis* (Okamura et al., 1993). With respect to its antibacterial properties, Altersolanol A was reported to interfere with the respiratory chains of bacterial membranes by acting as an electron acceptor (Haraguchi et al., 1992).

### Palmarumycin CP17 (58) and palmarumycin CP18 (59)

These new anti-parasitic natural products with pentacyclic spiroketal structures were isolated from an *Edenia* sp. (Pleosporaceae) fungus, obtained from mature leaves of *Petrea volubilis* (Verbenaceae), a tropical woody vine collected in Coiba National Park, Panama (Martínez-Luis et al., 2008). The compounds have an unusual structural feature involving two or three oxygen atoms which act as bridges between two original naphthalene subunits (Zhou et al., 2010a). These metabolites were shown to significantly inhibit growth of the amastigote form of the protozoan parasite *Leishmania donovani* (Martínez-Luis et al., 2008), a genus considered to be the second largest global parasitic killer of humans after malaria. The palmarumycins were also shown to have antineoplastic effects in mammalian cells by inhibiting the G_2_/M transition of the cell cycle through an unknown mechanism (Lazo et al., 2001).

### Rugulosin (60)

This bis-anthraquinoid pigment was isolated using extracts of *Hormonema dematioides*, an endophytic fungus of Canadian balsam fir trees, using bioassay-guided fractionation for inhibition of growth of spruce budworm (Calhoun et al., 1992). Conifer needles infected with the endophyte were associated with reduced weight gain of spruce budworm larvae when used as feed (Miller et al., 2002). Rugulosin has also been reported to be produced by various other fungal species including *Penicillium* (Ueno et al., 1980). Rugulosin is cytotoxic to both prokaryotes and eukaryotes and causes fatty degeneration, liver cell necrosis, and to a lesser extent hepatocarcinogenesis to mice and rats (Ueno et al., 1980). For these reasons, this compound is an important mycotoxin in yellow rice consumed in Asia (Chu, 1977).

### Nodulisporins

*Nodulisporium* sp. fungal endophytes (Xylariaceae) isolated from the endangered plant *Juniperus cedrus* (Canary Island Juniper, a gymnosperm) yielded nodulosporins A–C (**61–63**) which exhibited antifungal activity against *M. violaceum* (Dai et al., 2006). Interestingly, the same endophyte was isolated from an Angiosperm shrub, *Erica arborea*, from the Canary Island of Gomera from which related compounds were isolated called nodulisporins D–F (**64–66**) (Dai et al., 2009). Nodulisporins D–F showed antibacterial activity against *B. megaterium*, antifungal activity against *M. violaceum* (anther smut fungus), and anti-algal activity against *C. fusca*, using agar diffusion assays.

### Pyrrocidines A (67) and B (68)

These polyketide-amino acid-derived antibiotics were isolated from an endophyte of maize kernels, *Acremonium zeae*, a fungus which protects pre-harvest kernels against fungal pathogens, perhaps by competing for the same host niche in a temperature-dependent manner (Wicklow et al., 2005). Pyrrocidines displayed significant antifungal activity on agar disk assays against mycotoxin-producing *A. flavus* and *Fusarium verticillioides* (Wicklow et al., 2005). The authors reviewed previous studies which reported that pyrrocidine A inhibits growth of several Gram-positive bacteria. The study noted that *A. zeae* has sometimes been implicated as causing stalk rot and hence is selected against by breeders and pathologists, perhaps making commercial maize more susceptible to pathogens. In a subsequent study, pyrrocidine A showed potent activity against important ear rot and stalk rot pathogens of maize, including *Nigrospora oryzae*, *F. graminearum*, *Rhizoctonia zeae*, and *Stenocarpella* (*Diplodia*) *maydis* (Wicklow and Poling, 2009). The authors suggested that pyrrocidine A may protect vulnerable seedlings, in particular against pathogens, following colonization of the seedling by the endophyte from the seed. Pyrrocidine A also showed anti-pathogen activity against the seed-rot saprophytes *Eupenicillium ochrosalmoneum* and *A. flavus* as well as against the causal agent of fungal leaf spot disease, *Curvularia lunata*, and the bacteria *Clavibacter michiganense* subsp. *nebraskense*, the causative agent of Goss’s wilt (Wicklow and Poling, 2009). Other non-disease causing protective fungal endophytes were not as sensitive to pyrrocidines suggestive of evolutionary selection for fungal endophyte compatibility (Wicklow and Poling, 2009). However, pyrrocidin A showed antibiosis activity against two bacterial endophytes of maize used as biological control agents, *Bacillus mojavensis* and *Pseudomonas fluorescens* (Wicklow and Poling, 2009).

### Isofusidienol A–D (69–72)

These chromone-3-oxepine-polyketides were isolated from the fungus *Chalara* sp. (strain 6661), an endophyte of *Artemisia vulgaris*, an herb known as Mugwort which grows along the Baltic Sea coast. These compounds exhibited antifungal activity against the pathogenic yeast *C. albicans* and antibacterial activity against *B. subtilis*, *S. aureus*, and *E. coli* of which isofusidienol A was the most potent using agar disk assays (Lösgen et al., 2008).

### Chaetoglobosins A (73) and C (74)

These chlorinated azaphilone derivatives were characterized from cultures of the fungal endophyte *Chaetomium globosum* isolated from the leaves of *Ginkgo biloba*. These compounds exhibited significant toxicity toward brine shrimp larvae and antifungal activity against the industrial microbe *Mucor miehei* (Qin et al., 2009) a fungus used for the production of enzymes employed in the cheese industry.

### Chaetomugilin A (75) and D (76)

These azaphilone derivatives were also isolated from the fungal endophyte *C. globosum* as noted above. The compounds exhibited inhibitory activity against brine shrimp larvae (Qin et al., 2009).

### Pestalotheol C (77)

This compound was isolated from the endophytic fungus *Pestalotiopsis theae* which inhabits the branches of an unidentified tree in Hainan Province, China. In its pathogenic form, this fungus causes Tea Gray Blight disease. The compound showed inhibitory activity against HIV replication based on ELISA assays (Li et al., 2008b).

### CR377 (78)

This pentaketide was obtained from a *Fusarium* sp., an endophytic fungus living inside the stems of *Selaginella pallescens* (a resurrection plant given its ability to recover from severe dehydration), collected from the Guanacaste Conservation Area of Costa Rica. The compound exhibited antifungal activity against *C. albicans* using an agar diffusion assay with an inhibition zone similar to the fungicide nystatin (Brady and Clardy, 2000).

### Xanalteric acids I (79) and II (80)

These compounds were purified from an *Alternaria* sp. fungus, an endophyte isolated from the leaves of the Chinese mangrove plant *Sonneratia alba*. This plant is known as Mangrove Apple, an edible salt-tolerant plant eaten by humans and camels in Africa and the Pacific, but also used as a traditional herb against skin or intestinal parasites. These compounds exhibited weak antibiotic activity against *S. aureus* (Kjer et al., 2009).

### Pestalachloride B (81)

This compound was isolated from *P. adusta*, an endophytic fungus isolated from the stem of an unknown tree in China; the compound displayed significant antifungal activity against three important plant pathogens, *F. culmorum*, *G. zeae* (anamorph *F. graminearum*), and *V. albo-atrum* (Li et al., 2008a).

## Peptides

**Figure 8:**
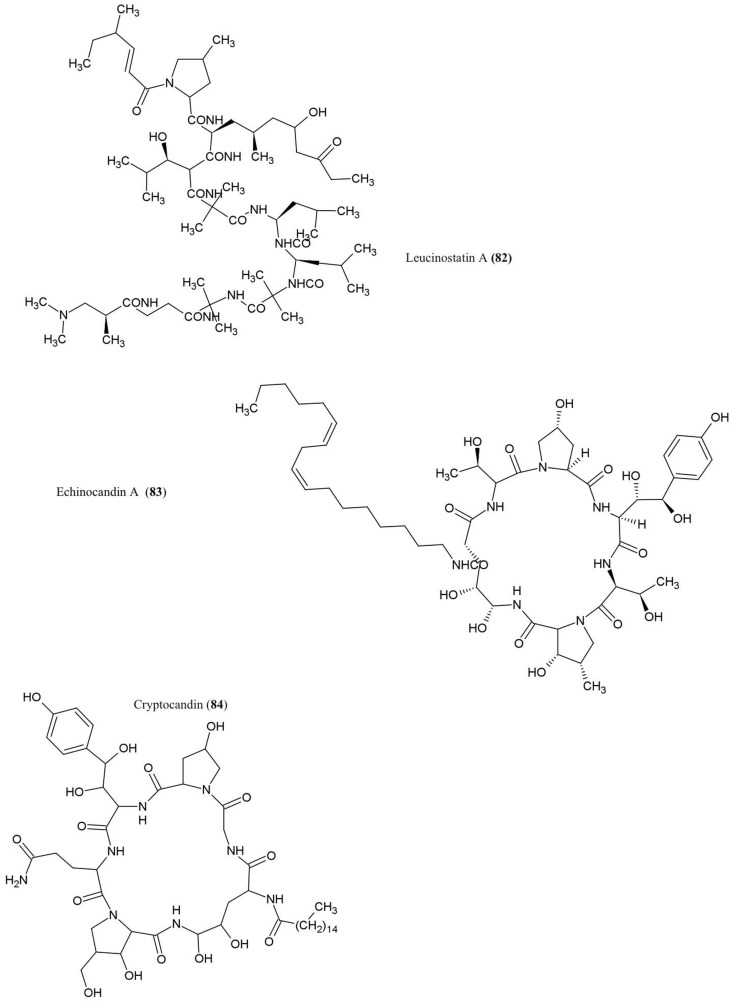
**Structures of peptides derivatives of fungal endophyte origin (82–84)**.

The structures of the peptide derivatives discussed in this review are illustrated (Figure 8).

### Leucinostatin A (82)

This compound was isolated from cultures of *Acremonium* sp., an endophytic fungus that colonizes *Taxus baccata* (European yew), an evergreen conifer tree. The compound was shown to have anti-cancer activities and could act as a fungicide against the oomycete *Pythium ultimum* (Strobel et al., 1997), an important plant pathogen that causes damping-off and root rot diseases.

### Echinocandin A (83)

This lipopetide was purified from cultures of the endophytic fungi *Cryptosporiopsis* sp. and *Pezicula* sp., inhabitants of *Pinus sylvestris* (Scots pine) and *F. sylvatica* (European beech), respectively. The compound showed antifungal activity against *C. albicans* and *Saccharomyces cerevisiae* (Noble et al., 1991). Mechanistically, echinocandins were found to inhibit the synthesis of cell wall glucans by inhibiting glucan synthase leading to cell lysis (Chapman et al., 2008).

### Cryptocandin (84)

This lipopeptide, related chemically to echinocandin, was isolated from the fungus *C*. *quercina*, an endophyte of the Chinese medicinal plant *Tripterigeum wilfordii* (Strobel et al., 1999). Cryptocandin was shown to have antifungal activity against multiple human pathogens including *C. albicans and Histoplasma capsulatum* (causal agent of the lung disease Histoplasmosis), in addition to *T. rubrum* and *T. mentagrophytes* (Strobel et al., 1999) – the latter two fungi cause skin and nail diseases in humans. The compound was also shown to inhibit the growth of phytopathogenic fungi including *Sclerotinia sclerotiorum*, the fungus that causes white mold disease which affects over 400 plant species, and *Botrytis cinerea*, a necrotic fungus that primarily affects grapes (Strobel et al., 1999).

## Conclusion and Future Directions

The objective of this paper was to review the diversity of secondary metabolites with anti-microbial activities produced by endophytic fungi, from the interdisciplinary perspectives of biochemistry, genetics, fungal biology, host plant biology, human and plant pathology. This review covered ∼80 compounds with diverse activities against plant and human pathogens, produced from a wide taxonomic diversity of endophytes inhabiting a range of plant species. Several major themes are apparent from the literature. With respect to biochemistry, fungal endophytes are able to produce almost all chemical classes of secondary metabolites, with terpenoids and polyketides being apparently the most common, and flavonoids and lignans being the rarest. Where endophytes have been investigated in depth biochemically such as *N. typhnium*, many compounds have been identified, suggesting that significant numbers of secondary metabolites remain to be discovered from less explored or unexplored endophytes. With respect to the genetic studies reviewed here, genes of fungal endophytes encoding anti-microbial secondary metabolites were observed to be clustered on chromosomes (e.g., loline alkaloids, ergot alkaloids, helvolic acid). Combined with the observation that the same secondary metabolite can be produced by different genera of endophytic fungi (e.g., paclitaxel, brefeldin A, echinocandin), this genetic clustering may have facilitated horizontal gene transfer of secondary metabolic pathways between fungal species during evolution. With respect to fungal biology, a complexity of this field of study is that fungi can exist in both sexual and asexual forms and can sometimes switch between endophytic and pathogenic lifestyles (e.g., *N. typhnium/E. typhina*), with each type potentially producing different classes of secondary metabolites. Finally, with respect to the biology of the host plants, angiosperms, gymnosperms, and lower plants were all found to be inhabited by fungal endophytes that could combat specific pathogens. However, treatment of crops with fungicides may be reducing natural fungicides by killing protective fungal endophytes of the host (e.g., pyrrocidine A produced from *A. zeae*). Often host plants that were investigated for endophyte-derived anti-microbial compounds were Chinese herbal medicinal plants or plants associated with indigenous knowledge concerning their anti-microbial properties (e.g., cryptocandin). Surprisingly, in some cases, both fungal endophytes and their host plants were reported to produce the same complex secondary metabolites, apparently redundantly (e.g., podophyllotoxin, paclitaxel). We conclude that fungal endophytes are potentially vital sources for natural products for agriculture, medicine, and industry, with significant potential to combat global crop and human pathogens which are becoming increasingly resistant to drugs and pesticides.

Despite the apparent progress, significant gaps remain in this field of research from an interdisciplinary perspective. Biochemically, many biosynthetic pathways and enzymes remain unidentified. How each endophyte and its host plant coordinate metabolic biosynthesis remains unexplored. For example, when both the plant and its endophyte produce the same secondary metabolite (e.g., paclitaxel), does the biosynthesis of this metabolite occur independently or is their signaling across organisms (e.g., feedback inhibition)? Furthermore, very little is known about the intracellular location of biosynthesis or mode of secretion of these anti-microbial compounds. Genetically, very few genes encoding the relevant biosynthetic enzymes have been isolated, and there is limited research as to how the expression of these genes is regulated at the molecular level, with only a few exceptions (e.g., Ergot alkaloids). Concerning fungal biology, factors which trigger the endophyte to change from mutualism to parasitism, along with associated changes in secondary metabolite production, remain poorly investigated. Concerning host plant biology, the literature appears to be biased for sampling endophytes from leaf, stem, and seed tissues compared to flowers, fruits, and roots. With respect to understanding the activities of these endophytes, there is limited information about structure-function relationships, specifically identifying moieties which can enhance and/or reduce the toxicity of the compound. Moreover, reducing the general toxicity of the compound may have positive effects on human health and natural ecosystems. Often anti-pathogen data is from *in vitro* studies only; however results from the natural environment may be different. Studies on anti-microbial activities of endophytes are often limited to a few model species including human pathogens with limited reports of plant viruses as targets. A better understanding of the contextual ecology of the host plant may be critical since identifying pathogens which inhabit the host may provide clues as to the specific anti-pathogenic targets of the endophyte under investigation (*A. zeae* produces pyrrocidine A to combat *A. flavus*). In conclusion, a more comprehensive understanding of the biochemistry, genetics and biology of endophyte and host, may lead to new opportunities for developing bio-based commercial products to combat global crop and human pathogens.

## Conflict of Interest Statement

The authors declare that the research was conducted in the absence of any commercial or financial relationships that could be construed as a potential conflict of interest.
